# Biological Activities of Essential Oils from Leaves of *Paramignya trimera* (Oliv.) Guillaum and *Limnocitrus littoralis* (Miq.) Swingle

**DOI:** 10.3390/antibiotics9040207

**Published:** 2020-04-24

**Authors:** Nhan Trong Le, Duc Viet Ho, Tuan Quoc Doan, Anh Tuan Le, Ain Raal, Donatella Usai, Giuseppina Sanna, Antonio Carta, Paola Rappelli, Nicia Diaz, Piero Cappuccinelli, Stefania Zanetti, Hoai Thi Nguyen, Matthew Gavino Donadu

**Affiliations:** 1 Faculty of Pharmacy, Hue University of Medicine and Pharmacy, Hue University, Hue 49000, Viet Nam; letrongnhan41@gmail.com (N.T.L.); hovietduc661985@gmail.com (D.V.H.); quoctuanqt91@gmail.com (T.Q.D.); 2 Mientrung Institute for Scientific Research, VAST, Hue 49000, Viet Nam; tasa207@gmail.com; 3 Institute of Pharmacy, Faculty of Medicine, University of Tartu, 50900 Tartu, Estonia; ain.raal@ut.ee; 4 Department of Biomedical Science, University of Sassari, 07100 Sassari, Italy; dusai@uniss.it (D.U.); rappelli@uniss.it (P.R.); ndiaz@uniss.it (N.D.); zanettis@uniss.it (S.Z.); mdonadu@uniss.it (M.G.D.); 5 Department of Biomedical Science, University of Cagliari, 09042 Monserrato, Italy; g.sanna@unica.it; 6 Department of Chemistry and Pharmacy, University of Sassari, 07100 Sassari, Italy; acarta@uniss.it

**Keywords:** antifungal activity, antibacterial activity, infections, essential oils

## Abstract

The present study aimed to determine the bioactivities of essential oils extracted from the leaves of *Paramignya trimera* and *Limnocitrus littoralis*, including cytotoxicity, antiviral, antibacterial, antimycotic, and antitrichomonas effects. Herein, it was indicated that *P. trimera* and *L. littoralis* oils showed no cytotoxicity on normal cells, namely MT-4, BHK-21, MDBK, and Vero-76. *P. trimera* oil (i) exhibited the strongest inhibition against *Staphylococcus aureus* with MIC and MLC values of 2% (*v/v*); (ii) showed MIC and MLC values of 8% (*v/v*) in *Candida parapsilosis*; and (iii) in the remaining strains, showed MIC and MLC values greater than or equal to 16% (*v/v*). On the other hand, *L. littoralis* oil (i) displayed the strongest inhibition against *Candida tropicalis* and *Candida parapsilosis* with 2% (*v/v*) of MIC and MLC; and (ii) in the remaining strains, possessed MIC and MLC greater than or equal to 16% (*v/v*). In addition, antitrichomonas activities of the oils were undertaken, showing IC_50_, IC_90_, MLC values, respectively, at 0.016%, 0.03%, and 0.06% (*v/v*) from *P. trimera,* and 0.03%, 0.06%, 0.12% (*v/v*) from *L. littoralis*, after 48 h of incubation. The oils were completely ineffective against ssRNA+ (HIV-1, YFV, BVDV, Sb-1, CV-B4), ssRNA- (RSV, VSV), dsRNA (Reo-1), and dsDNA (HSV-1, VV) viruses. This is the first report describing the cytotoxicity, antiviral, antibacterial, antimycotic, and antitrichomonas activities of the essential oils of *P. trimera* and *L. littoralis*.

## 1. Introduction

Essential oils, also known as volatile oils or ethereal oils, are aromatic oily liquids extracted from different parts of the plants [1] and contain a wide range of chemical compounds, mainly terpenes and terpenoids [2]. The number of identified essential oils has reached 3000, and most have been used in many applications, namely pharmaceuticals, fragrances, and the food industry [3]. Some of these have been used to treat certain organ and systemic disorders [4]. Essential oils exhibit powerful antibacterial, antifungal, antioxidant, antiviral, anti-inflammatory, and anticancer properties [5,6,7]. A plethora of active constituents with high biological values have been found in essential oils, especially such possessing antimicrobial characteristics [8,9].

The *Paramignya* genus is a member of the Rutaceae family, including 28 species. The extraction of the *Paramignya* species comprised a wide range of chemical constituents such as coumarins, triterpenes, alkaloids, and glycoside derivatives. The *Paramignya* species were employed in traditional medication treating cancer, hepatitis, diabetes and nose infections. Particularly, their extracted-active compounds exhibited anti-tumour, antioxidant, and anti-inflammatory effects [10]. However, to date, there have not been many attempts on investigating the chemical compositions and biological activities of essential oils extracted from these species. *Paramignya trimera* (Oliv.) Guillaum (*P. trimera*) is a woody species and is widely distributed in the South of Vietnam [11]. The stems and roots have been traditionally applied in the treatment of liver illness and diabetes [12]. Our previous research indicated that β-caryophyllene (10.5%), β-caryophyllene oxide (9.9%), 7-epi-α-eudesmol (7.6%), and γ-muurolene (6.8%) were major constituents of the *P. trimera* essential oil [12].

The genus *Limnocitrus* includes the only known species *Limnocitrus littoralis* (Miq.) Swingle (*L. littoralis*) and is known as an endangered species in the Red List of the International Union for Conservation of Nature [13]. *L. littoralis* Swingle is a knotty shrub species and is naturally distributed in the coastal areas of Indonesia and the Middle and South of Vietnam [13]. In Vietnamese traditional medicine, the parts of *L. littoralis* have been utilised to treat fevers and colds and have antipyretic and antitussive effects [14]. The extraction of *L. littoralis* has been used in cosmetics to reduce non-pathological skin appearances of inflammatory origin [14]. We identified the main components of *L. littoralis* essential oil such as myrcene (24.9%), γ–murolene (11.0%), and oleic acid (10.3%) [14]. 

Along these lines, in order to give additional information about biological activities of essential oils from *P. trimera* and *L. littoralis* collected in Vietnam, we report for the first time the biological activities of the essential oils from the leaves of *P. trimera* and *L. littoralis*. The aim of this study is to determine the cytotoxicity, antiviral, antibacterial, and antimycotic and antitrichomonas effects of essential oils extracted from the leaves of *P. trimera* and *L. littoralis*. 

## 2. Results

### 2.1. Cytotoxicity and Antiviral Activity

Herein, we explored antiviral properties of essential oils extracted from leaves of *P. trimera* and *L. littoralis* against RNA and DNA viruses of several important human pathogens. First, the studied ssRNA+ viruses included the following: human immunodeficiency virus type-1 (HIV-1) (Retroviridae); two Flaviviridae, bovine viral diarrhoea virus (BVDV) and yellow fever virus (YFV); and two Picornaviridae, human enterovirus B (coxsackie virus B4, CV-B4) and human enterovirus C (polio virus type-1, Sb-1). Second, the ssRNA- viruses in this case contained human respiratory syncytial virus (hRSVA2) (Pneumoviridae) and vesicular stomatitis virus (VSV) (Rhabdoviridae). Third, among dsRNA viruses, we tested reovirus type-1 (Reo-1) (Reoviridae). Finally, representatives of two DNA virus families were also included: vaccinia virus (VV) (Poxviridae) and human herpes virus 1 (herpes simplex type-1, HSV-1) (Herpesviridae). In order to understand their selective antiviral activities, the cytotoxicity tests were undertaken in parallel assays with uninfected cell lines, including MT-4 cells (CD4+ human T-cells containing an integrated HTLV-1 genome), Madin Darby Bovine Kidney (MDBK) (ATCC CCL 22 (NBL-1) Bos taurus), Baby Hamster Kidney (BHK-21) (ATCC CCL 10 (C-13) Mesocricetus auratus); Monkey kidney (Vero-76) (ATCC CRL 1587 Cercopithecus Aethiops). In vitro cytotoxicity was measured based on cell proliferation and viability. The CC_50_ (drug concentration inhibiting cell growth by 50% referred to untreated control) was >100 µg/mL, and no cell toxic effect was observed. However, results obtained from our screening showed that *P. trimera* and *L. littoralis* essential oils were completely ineffective against the tested viruses with EC_50_ values over 100 µg/mL (Table 1).

### 2.2. Antimicrobial Activities

The antimicrobial activities of essential oils from *P. trimera* and *L. littoralis* are displayed in Table 2. In general, Gram-positive bacteria were more sensitive to *P. trimera* oil than Gram-negative bacteria, whereas *L. littoralis* oil did not show any significant activity on both Gram-positive and Gram-negative strains. For *Candida* species, *P. trimera* and *L. littoralis* oils indicated the best antimicrobial activity against *Candida parapsilosis (C. parapsilosis*). Particularly, for *P. trimera* oil, i) it exhibited strongest inhibition against *Staphylococcus aureus* (*S. aureus*) with Minimum Inhibitory Concentrations (MIC) and Minimum Lethal Concentrations (MLC) values of 2% (*v/v*); ii) it showed MIC and MLC values of 8% (*v/v*) in *C. parapsilosis*; iii) the remaining strains had MIC and MLC values greater than or equal to 16% (*v/v*). For *L. littoralis* oil, i) it displayed strongest inhibition against *Candida tropicalis* (*C. tropicalis*) and *C. parapsilosis* with 2% (*v/v*) MIC and MLC; ii) the remaining strains possessed MIC and MLC more than or equal to 16% (*v/v*). The microbicidal or microbiostatic action is indicated via the ratio of MLC/MIC, whose value less than or equal to 4.0 shows a microbicidal effect, whilst that greater than 4.0 shows that it exerts a microbiostatic action [15,16]. Herein, the MLC/MIC ratios obtained from the experimental data were all equal to 1, proving that both *P. trimera* and *L. littoralis* essential oils showed bactericidal and fungicidal properties on the studied strains. 

### 2.3. Antitrichomonas Activity

As shown in Table 3, both tested essential oils have a cytotoxic activity against *Trichomonas vaginalis* (*T. vaginalis*). It can be observed that the values of 50% inhibitory concentration (IC_50_), ≥90% inhibitory concentration (IC_90_) and MLC values against *T. vaginalis* were time-dependent in the given period. Essential oils showed a weak effect after 1 h of incubation, which promptly increased over time. Both *P. trimera* and *L. littoralis* essential oils strongly affected *T. vaginalis* viability at 24 h with a 64- and 32-fold increase, respectively, compared to 1 h. At 48 h, the antitrichomonas activity of *P. trimera* and *L. littoralis* oils increased 2 times and 4 times more than those of 24 h, respectively. In particular, the leaves-extracted oils had IC_50_, IC_90_, and MLC values, respectively, at 0.016%, 0.03%, and 0.06% (*v/v*) from *P. trimera,* and 0.03%, 0.06%, 0.12% (*v/v*) from *L. littoralis* after 48 h. 

## 3. Discussion

Essential oils comprise a myriad of more than 20 active molecules with variable low molecular weight at distinct concentrations [17]. Essential oils can exert antimicrobial properties thanks to the complex interactions between their constituents, including phenols, alcohols, aldehydes, ketones, esters, ethers, and hydrocarbons [17], or being associated with their major components [18,19]. Each substituent of the essential oil displays a variety of mechanisms of action or cellular pathways against microorganisms [1] and modulates the effects of other components present in the same essential oil [17]. Therefore, a broad range of different chemical compositions in the essential oils are likely to result in their disparate biological properties. As an example, essential oils bearing high amounts of phenolic derivatives such as carvacrol and thymol were demonstrated to exhibit strong antimicrobial effect against bacteria [1]. In addition, the abundant oxygenated monoterpenes in essential oils were reported to show higher antibacterial effect compared to those with monoterpene hydrocarbons [20]. The essential oil of *P. trimera* consisted of two main classes: oxygenated sesquiterpenes (41.7%) and sesquiterpene hydrocarbons (39.6%) with major constituents, namely β-caryophyllene (10.5%), β-caryophyllene oxide (9.9%), 7-epi-α-eudesmol (7.6%), and γ-muurolene (6.8%) [12]. On the other hand, the main compounds of *L. littoralis* oil were classified into two main categories: sesquiterpene hydrocarbons (32.3%) and monoterpene hydrocarbons (27.7%), in which myrcene (24.9%), γ-muurolene (11.0%), and oleic acid (10.3%) were found as major constituents [14]. In the present work, it can be seen that the differences of chemical compositions between *P. trimera* and *L. littoralis* essential oils, especially their main constituents ultimately induced the distinct antimicrobial activities. 

Among the above-studied bacteria, the *P. trimera* essential oil showed enhanced sensitivity in Gram-positive bacteria than Gram-negative ones. In fact, Gram-negative bacteria are made up of more complex cell walls than those of Gram-positive bacteria, thus rendering Gram-positive bacteria more susceptible to penetration of the essential oil to inhibit the bacteria [21,22]. Herein, we indicated that the essential oil of *P. trimera* was more effective against *S. aureus* compared to *Enterococcus faecalis*, while *L. littoralis* essential oil did not show any activity towards Gram-positive bacteria. This is hypothetically attributed to the difference in chemical compositions of these oils. The antibacterial activity of *P. trimera* essential oil against *S. aureus* was likely due to its major compounds such as β-caryophyllene and β-caryophyllene oxide. According to Dahham et al. [23], β-caryophyllene from *Aquilaria crassna* essential oil inhibited *S. aureus* with an MIC value of 3 ± 0.4 (µM). Ali et al. [24] demonstrated that *Teucrium yemense* essential oil contains high levels of β-caryophyllene and that caryophyllene oxide has the ability to inhibit *S. aureus* with an MIC value of 0.156 mg/mL.

*S. aureus* is one of the most common causes of nosocomial infections and postsurgical wound contamination. It is also considered as one of the main etiologic contributions in food-related infections. Moreover, the development of *S. aureus*-resistant methicillin has gained rapidly growing attention over the last decade [25]. In the context of traditional medicine research, some studies have focused on investigating the efficacy of some essential oils against both Methicillin-sensitive and Methicillin-resistant *S. aureus*. It was reported that *Melaleuca alternifolia* oil was effective against *S. aureus* with MIC_90_ of 0.5% (*v/v*) and Methicillin-resistant *S. aureus* with MIC_90_ at 0.32% (*v/v*) [26]. Kwiatkowski et al. [27] indicated that *Carum carvi, Pogostemon cablin,* and *Pelargonium graveolens* essential oils showed efficiency against *S. aureus* with MIC values of 1.88 ± 1.03, 0.17 ± 0.08, and 0.54 ± 0.20% (*v/v*), respectively. The growth of the standard and clinical isolates of Methicillin-resistant *S. aureus* and Methicillin-sensitive *S. aureus* were inhibited by *Zataria multiflora* essential oil at concentrations of 0.55 to 1.41 µL/mL [25]. On the other hand, in the current work, *P. trimera* oil displayed inhibition against *S. aureus* with MIC and MLC of 2% (*v/v*) and exhibited no cell toxic effect at the same concentration, and thus *P. trimera oil* could be used safely at this dose. However, further testing needs to be done to study the mutagenicity, teratogenicity, or other side effects before considering using it as an agent against *S. aureus*.

*Candida* species are involved in mucocutaneous infections and are considered as one the most common causes of blood-stream infections. However, over the last decade, some clinical isolates of *Candida* species have been developing great resistance against the conventional use of triazole antifungal drugs such as itraconazole and fluconazole; therefore, the demand for newly effective antifungals is urgently needed [25]. Indeed, essential oils can be employed as anti-*Candida* agents against azole-resistant strains [28]. Vishnu et al. [28] investigated 30 essential oils against *C. albicans*, of which 18 were found to be effective; particularly eucalyptus and peppermint oils were proved to be the most effective. Nidhi et al. [29] reported the antimicrobial activities of olive and cinnamon oils on 100 *Candida* isolates, and around 50% of these *Candida* species exhibited sensitivity against the two studied oils. *C. tropicalis* and *C. parapsilosis* were more sensitive to the *L. littoralis* oil compared to the other *Candida* species tested, and *C. parapsilosis* was more sensitive to the *P. trimera* oil compared to the other Candida species in the present study. Given the experimental data obtained in this case and our previous works [30,31], the resistance may vary depending on the species of *Candida* and the essential oils. The current work indicated that *L. littoralis* oil was effective against *C. tropicalis* and *C. parapsilosis* with MIC and MLC of 2% (*v/v*), while exerting no cytotoxicity at the same concentration. Therefore, *L. littoralis* essential oil can be used as a potent anti-*Candida* agent on *C. tropicalis* and *C. parapsilosis*. 

*T. vaginalis* is a protozoan parasite and is infective agent in human vagina, prostate gland, and urethra [32]. *T. vaginalis* is a flagellated parasite affecting about 156 million people each year in the world [32]. Trichomoniasis infection may cause serious health consequences, especially for women [33]. The current treatment is being based upon 5-nitroimidazole [33]. However, the emergence of resistance has limited the effectiveness of this therapy [34,35]. Therefore, it is of critical importance to search for novel pharmaceutical alternatives to treat trichomoniasis successfully in clinical settings [36]. Only a few essential oils, especially some species of the Lamiaceae family, have been investigated against *Trichomonas* [32,36,37,38,39]. Particularly, the essential oil of *Marrubium vulgare* displayed antitrichomonas activity with an average MIC value of 291 ± 136 µg/mL after 48 h of incubation [36]. Hayam et al. [37] investigated the in vitro effects of *Ocimum basilicum* essential oil on *T. vaginalis* trophozoites and showed its MLC values of 30, 20, and 10 µg/mL after 24 h, 48 h, and 96 h of incubation, respectively. The previous study has demonstrated that low (≤1%) concentrations of two *Lavandula* essential oils (*L. angustifolia* and *L. intermedia*) can completely eliminate *T. vaginalis* in vitro [39]. After *Amomum tsao-ko* essential oil treatment, a series of modifications were observed in *T. vaginalis* cells. For example, nuclear membrane was disrupted, nuclei were dissolved, ribosomes were reduced, rough endoplasmic reticulum dilated, various vacuoles appeared, and organelles disintegrated. Furthermore, the cell membrane was partially damaged, and a leak of cytoplasmic led to cell disintegration [40]. In this work, we observed a prompt cytopathic activity of *P. trimera* and *L. littoralis* essential oils against *T. vaginalis*. Our results suggest further investigation since they could represent promising trichomonacidal agents.

Multidrug resistance is of great concern around the world and has caused a large number of deaths worldwide over the last decade [41]. Every day, bacteria and viruses have been advancing their resistance mechanisms against our current anti-infectious medications at a dramatic pace, making it exceptionally difficult to cope with given the available tools [34,35,42,43]. Furthermore, the use of synthetic chemicals to control microorganisms is facing several limitations due to their carcinogenic effects, acute toxicity, and environmental hazards [2]. As a result, it is of utmost importance to design novel antibiotics with high efficiency and non-toxicity [21]. To this end, plant-based therapeutic agents have gained extensive attention as a potential natural source for treating infectious diseases over the last few years [31,40,44]. For this reason, the present work, for the first time, discussed the cytotoxicity, antiviral, antibacterial, antimycotic, and antitrichomonas effects of essential oils from *P. trimera* and *L. littoralis*, showing great activity against *S. aureus*, *C. tropicalis*, *C. parapsilosis,* and *T. vaginalis.* Further research is needed on the biological effects as well as the toxicity of *P. trimera* and *L. littoralis* essential oils, before considering using them as therapeutic agents to treat infectious diseases.

## 4. Materials and Methods 

### 4.1. Plant Material 

The leaves of *P. trimera* and *L. littoralis* were collected from Hue and Quang Ngai provinces, Vietnam, respectively, in May 2019. Plant samples were identified by Dr. Chinh Tien Vu, Vietnam National Museum of Nature. Two voucher specimens (TTH-T110 and QNG-T112) were deposited at the Faculty of Pharmacy, Hue University of Medicine and Pharmacy, Vietnam.

### 4.2. Extraction of the Essential Oils

The leaves of *P. trimera* and *L. littoralis* were shredded, and the essential oils were hydrodistilled for 3.5 h at ambient pressure using a Clevenger-type apparatus [45]. The oils were dried on Na_2_SO_4_ and stored in sealed vials, at 4 °C, ready for the biological activities test.

### 4.3. Cells and Cytotoxicity Assays

All cells were purchased from American Type Culture Collection (ATCC). MT-4 cells (CD4+ human T cells containing an integrated HTLV-1 genome) were cultured in Roswell Park Memorial Institute 1640 (RPMI-1640) medium supplemented with 10% fetal bovine serum (FBS), 100 units/mL penicillin G, and 100 μg/mL streptomycin; Madin Darby Bovine Kidneys (MDBK) (ATCC CCL 22 (NBL-1) Bos taurus) were cultured in Minimum Essential Medium with Earle’s salts (MEM-E), L-glutamine, 1mM sodium pyruvate and 25mg/L kanamycin, supplemented with 10% horse serum; Baby Hamster Kidneys (BHK-21) (ATCC CCL 10 (C-13) Mesocricetus auratus) were cultured in (MEM-E), L-glutamine, 1mM sodium pyruvate and 25 mg/L kanamycin, supplemented with 10% FBS; and Monkey kidneys (Vero-76) (ATCC CRL 1587 Cercopithecus Aethiops) were cultured in in Dulbecco’s Modified Eagle Medium (D-MEM) with L-glutamine and 25 mg/L kanamycin, supplemented with 10% FBS. Cells were checked regularly for the absence of mycoplasma contamination with MycoTect Kit (Gibco).

MT-4, BHK-21, MDBK, and Vero-76 cells were seeded at 4 × 10^5^ cells/mL, 6 × 10^5^ cells/mL, 1 × 10^6^ cells/mL, and 5 × 10^5^ cells/mL, respectively. Cell cultures were treated with varying concentrations of essential oils ranging from 0.8–100 μg/mL. Equivalent DMSO concentration were also added as control and then incubated at 37 °C in a humidified, 5% CO_2_ atmosphere. Cell viability was determined at 37 °C by the 3-(4,5-dimethylthiazol-1-yl)-2,5-diphenyltetrazolium bromide (MTT) method [46]. The test medium used for the cytotoxic assay as well as for antiviral assay contained 1% of the appropriate serum. DMSO was used as control in each experiment, and it was tested at the maximum concentration present in each compounds. The cytotoxicity of test compounds was evaluated in parallel with their antiviral activity.

### 4.4. Viruses and Antiviral Assays

All viruses were purchased from American Type Culture Collection (ATCC), with the exclusion of Human Immunodeficiency Virus type-1 (HIV-1) and Yellow Fever Virus (YFV): IIIB laboratory strain of HIV-1 was obtained from the supernatant of the acutely infected H9/IIIB cells (NIH 1983); yellow fever virus (YFV) (strain 17-D vaccine (Stamaril Pasteur J07B01)); bovine viral diarrhoea virus (BVDV) (strain NADL (ATCC VR-534)); coxsackie type B4 (CV-B4) (strain J.V.B. (ATCC VR-184)); human enterovirus C (poliovirus type-1 (Sb-1) (Sabin strain Chat (ATCC VR-1562)); vesicular stomatitis virus (VSV) (lab strain Indiana (ATCC VR 1540)); human respiratory syncytial virus (hRSV) (strain A2 (ATCC VR-1540)); reovirus type-1 (Reo-1) (simian virus 12, strain 3651 (ATCC VR- 214)), vaccinia virus (VV) (vaccine strain Elstree-Lister (ATCC VR-1549)); human herpes 1 (HSV-1) (strain KOS (ATCC VR-1493)).Viruses were maintained in our laboratory and propagated in suitable cell lines. The viruses were stored in small aliquots at −80 °C until use.

Essential oil’s activity against HIV-1 was evaluated as follow: 50 μL of RPMI-1640 containing 1 × 10^4^ MT-4 cells were added to each well of flat-bottom microtitre trays, containing 50 μL of RPMI-1640 with or without serial dilutions of the essential oils. Then, 20 μL of an HIV-1 suspension containing 100 CCID_50_ was added. Essential oil’s activity against YFV and Reo-1 was based on inhibition of virus-induced cytopathogenicity in BHK-21 cells acutely infected at an multiplicity of infection (m.o.i) of 0.01. Essential oil’s activity against BVDV was based on inhibition of virus-induced cytopathogenicity in MDBK cells acutely infected at an m.o.i. of 0.01. Briefly, BHK and MDBK cells were seeded in 96-well plates at a density of 5 × 10^4^ and 3 × 10^4^ cells/well, respectively, and were allowed to form confluent monolayers by incubating overnight in growth medium at 37 °C in a humidified CO_2_ (5%) atmosphere. Cell monolayers were then infected with 50 μL of a proper virus dilution in MEM-E, then 50 μL of medium, with or without serial dilutions of the essential oils. After a 3- or 4-day incubation at 37 °C, cell viability was measured by the MTT method [46].

Activities of essential oils and compounds against CV-B4, Sb-1, VV, VSV, hRSV A2, and HSV-1 was determined by plaque reduction assays in infected cell monolayers as described previously [47]. Briefly, Vero-76 cells were cultured in 24-well tissue culture plate and with approximately 50/100 PFU/well of virus. After 2 h on a rocker, the unadsorbed viruses were removed and replaced with 500 μL of D-MEM containing 0.75% methyl-cellulose, with serial dilutions of test products. The overlayed medium was also added to not treat wells as non-infection controls. Cultures were incubated at 37 °C and then fixed with PBS containing 50% ethanol and 0.8% crystal violet, washed and air-dried. The number of plaques in the control (no inhibitor) and experimental wells were then counted.

### 4.5. Antimicrobial Activities

In the present work, a collection of 12 strains were selected, including 5 Gram-negative strains (*Escherichia coli* ATCC 35218, *Escherichia coli* clinical, *Pseudomonas aeruginosa* ATCC 27853, *Pseudomonas aeruginosa* clinical and *Klebsiella pneumoniae* clinical); 3 Gram-positive strains (*Staphylococcus aureus* ATCC 43300, *Staphylococcus aureus* clinical and *Enterococcus faecalis* clinical); and 4 *Candida* species strains (*Candida albicans* 556 RM, *Candida glabrata* clinical, *Candida tropicalis* 1011 RM and *Candida parapsilosis* RM). Cultures were maintained in appropriate media at 4 °C. The cells were cultivated at 37 °C on agar plates for 18 h prior to experiments.

### 4.6. Determination of Minimum Inhibitory Concentrations (MIC) and Minimum Lethal Concentration (MLC)

In order to establish the MIC and MLC of bacteria and *Candida* species, the broth dilution method was employed as reported by the Clinical and Laboratory Standard Institute [48]. The inoculum was prepared by diluting colonies in salt solution at a concentration of 0.5 McFarland, then confirmed at a wavelength of 530 nm by a spectrophotometric reading. The sensitivity test was implemented in Luria Bertani broth and RPMI-1640 medium using 96-well plates. The oil solutions were diluted to a range of concentrations from 16% (*v/v*) to 5 × 10^−4^% (*v/v*). After shaking, 100 μL of each oil dilution and 100 μL of bacterial/yeast suspension at a concentration of 10^6^ CFU/mL were added to each well and then incubated within 24 h for bacteria and 48 h for fungi at 37 °C. MIC values were determined by the lowest concentration of the essential oils in which bacterial growth is visibly inhibited after overnight incubation. In order to determine the MLC values, 10 μL was seeded on Mueller Hinton agar and Sabouraud Dextrose agar and the plates were incubated within 24 h for bacteria and 48 h for fungi at 37 °C. Minimal Lethal Concentration (MLC) was considered as the lowest concentration that reduces the viability of the initial microbial inoculum by ≥99.9%. A positive growth control consisting of organisms in broth and a negative sterility control consisting of uninoculated broth were included for each assay. Each experiment was performed in duplicate and repeated three times.

### 4.7. Antitrichomonas Activity 

*Trichomonas vaginalis* were cultured axenically in vitro by daily passages in Diamond’s Trypticase Yeast extract Maltose (TYM) medium (Sigma Chemical Co., St. Louis, MO, USA) plus 10% FBS (GIBCO, Invitrogen) at 37 °C in a 5% carbon dioxide atmosphere [49]. Exponentially growing *T. vaginalis* cells were harvested and viability was assessed by microscopy. Trichomonas cells (viability >95%) were centrifuged at 1500 rpm for 10 min and resuspended in Diamond’s TYM medium at a concentration of 2 × 10^5^ cells/mL [50].

Essential oils from *P. trimera* and *L. littoralis* were serially diluted in 100 μL of Diamond’s TYM medium from 16% to 0.002% (*v/v*) in 96-well plates, and 100 μL of the prepared Trichomonas suspension was added to each well. Diamond’s TYM medium alone was used as a growth control. The culture plate was kept at 37 °C in a CO_2_ incubator and checked after 1, 4, 24, and 48 h. The percentage of viable *T. vaginalis* cells was assessed by microscopic observation. The MLC was defined as the lowest essential oils concentration in which no viable protozoa were observed. The IC_50_ and IC_90_ values were considered as the oils concentration in which 50% and ≥90% *T. vaginalis* cells were killed. Each assay has been repeated independently at least two times [40].

### 4.8. Linear Regression Analysis and Statistical Analysis

The extent of cell growth/viability and viral multiplication, at each drug concentration tested, were expressed as percentage of untreated controls. Concentrations resulting in 50% inhibition (CC_50_ or EC_50_) were determined by linear regression analysis.

Data were processed for ANOVA by means of the software MSTAT-C, and mean separation was performed by application of the LSD test at *p* ≤ 0.05 level of significance.

## 5. Conclusions

The biological activities of *P. trimera* and *L. littoralis* essential oils were determined for the first time. Both oils displayed no cytotoxicity on normal cells such as MT-4, BHK-21, MDBK, and Vero-76. The *P. trimera* oil displayed the strongest sensitivity against *S. aureus*, whereas that of *L. littoralis* exhibited the strongest inhibition towards *C. tropicalis* and *C. parapsilosis*, with MIC and MLC values of 2% (*v/v*). In addition, *P. trimera* essential oil inhibited *C. parapsilosis* as observed in MIC and MLC of 8% (*v/v*). The oils also demonstrated inhibition against *T. vaginalis* with IC_50_, IC_90_, and MLC, respectively, at 0.016%, 0.03%, and 0.06% (*v/v*) from *P. trimera* and 0.03%, 0.06%, and 0.12% (*v/v*), respectively, from *L. littoralis* after 48 h of incubation. These studied essential oils were ineffective against HIV-1, YFV, BVDV, Sb-1, CV- B4, RSV, VSV, Reo-1, HSV-1, and VV viruses. Further studies should be implemented to evaluate the safety and toxicity of *P. trimera* and *L. littoralis* essential oils in humans before considering the development new anti-infectious agents for use in clinical trials.

## Figures and Tables

**Table 1:** Cytotoxicity and antiviral activity of essential oils from *P. trimera* and *L. littoralis* against representatives of ssRNA^+^ (HIV-1, YFV, BVDV, Sb-1, CV- B4), ssRNA^−^ (RSV, VSV), dsRNA (Reo-1), and dsDNA (HSV-1, VV) viruses.

Cell Lines and Virus	MT-4	HIV-1_IIIB_	BHK-21	YFV	Reo-1	MDBK	BVDV	Vero-76	RSV	VSV	HSV-1	VV	Sb-1	CV-B4
	CC_50_^a^	EC_50_^b^	CC_50_^c^	EC_50_^d^	EC_50_^d^	CC_50_^e^	EC_50_^f^	CC_50_^g^	EC_50_^h^					
O.P.t	>100	>100	>100	>100	>100	>100	>100	>100	>100	>100	>100	>100	>100	>100
O.L.l	>100	>100	>100	>100	>100	>100	>100	>100	>100	>100	>100	>100	>100	>100
* Reference Compound														
RC1	40	0.003 ± 0.0003	-	-	-	-	-	-	-	-	-	-	-	-
RC2	-	-	80	1.4 ± 0.2	-	>100	1.7 ± 0.3	-	-	-	-	-	-	-
RC3	-	-	-	-	-	-	-	>100	-	-	-	-	2	2 ± 0.5
RC4	-	-	-	-	-	-	-	≥14	2 ± 0.2	-	-	-	-	-
RC5	-	-	-	-	-	-	-	>100	-	-	3.0 ± 0.1	-	-	-
RC6	-	-	-	-	-	-	-	>100	-	-	-	1.7 ± 0.1	-	-
RC7	-	-	>100	-	17	-	-	-	-	-	-	-	-	-

Data represent mean values + standard deviation (SD) for three independent determinations. For values where SD is not shown, variation among duplicate samples was less than 15%. O.P.t: essential oil from the leaves of *Paramignya* *trimera;* O.L.l: essential oil from the leaves of *Limnocitrus* *littoralis*; RC1: Efavirenz; RC2: 2′-C-methylguanosine; RC3: Pleconaril; RC4: 6-aza-uridine; RC5: Acycloguanosine; RC6: Mycophenolic acid; RC7: 2′-C-methylcytidine. ^a^ Compound concentration (µg/mL) required to reduce the proliferation of mock-infected MT-4 cells by 50%, as determined by the 3-(4,5-dimethylthiazol-1-yl)-2,5-diphenyltetrazolium bromide (MTT) method. ^b^ Compound concentration (µg/mL) required to achieve 50% protection of MT-4 cells from HIV-1-induced cytopathogenicity, as determined by the MTT method. ^c^ Compound concentration (µg/mL) required to reduce the viability of mock-infected Baby Hamster Kidney (BHK) cells by 50%, as determined by the MTT method. ^d^ Compound concentration (µg/mL) required to achieve 50% protection of BHK cells from yellow fever virus (YFV) or Reo-1 induced cytopathogenicity, as determined by the MTT method. ^e^ Compound concentration (µg/mL) required to reduce the viability of mock-infected Madin Darby Bovine Kidney (MDBK) cells by 50%, as determined by the MTT method. ^f^ Compound concentration (µg/mL) required to achieve 50% protection of MDBK cells from bovine viral diarrhoea virus (BVDV)-induced cytopathogenicity, as determined by the MTT method. ^g^ Compound concentration (µg/mL) required to reduce the viability of mock-infected Vero-76 cells by 50%, as determined by the MTT method. ^h^ Compound concentration (µg/mL) required to reduce the plaque number of RSV, VSV, HSV-1, VV, Sb-1, and CV-B4 by 50% in Vero-76 monolayers. * Reference compound: CC_50_ and EC_50_ are in µM.

**Table 2:** Antimicrobial activities (MIC and MLC) of essential oils from the leaves of *P. trimera* and *L. littoralis.*

Strains	*P. trimera* Oil		*L. littoralis* Oil	
	MIC (% *v/v*)	MLC (% *v/v*)	MIC (% *v/v*)	MLC (% *v/v*)
**Gram-Positive Bacteria**				
*Staphylococcus aureus* ATCC 43300	2 ± 0.5	2 ± 0.5	>16 ± 0.5	>16 ± 0.5
*Staphylococcus aureus* clinical strain	2 ± 0.5	2 ± 0.5	>16 ± 0.5	>16 ± 1
*Enterococcus faecalis* clinical strain	16 ± 0.5	16 ± 1	>16 ± 1	>16 ± 1
**Gram-Negative Bacteria**				
*Escherichia coli* ATCC 35218	>16 ± 1	>16 ± 1	>16 ± 1	>16 ± 0.5
*Escherichia coli* clinical strain	>16 ± 1	>16 ± 0.5	>16 ± 0.5	>16 ± 0.5
*Pseudomonas aeruginosa* ATCC 27853	16 ± 0.5	>16 ± 1	>16 ± 1	>16 ± 1
*Pseudomonas aeruginosa* clinical strain	16 ± 0.5	>16 ± 0.5	>16 ± 0.5	>16 ± 1
*Klebsiella pneumoniae* clinical strain	>16 ± 0.5	>16 ± 0.5	>16 ± 0.5	>16 ± 1
**Yeast**				
*Candida albicans* 556 RM	16 ± 1	16 ± 0.5	16 ± 0.5	16 ± 1
*Candida glabrata* clinical	16 ± 1.5	16 ± 1	>16 ± 0.5	>16 ± 0.5
*Candida tropicalis* 1011 RM	16 ± 0.5	16 ± 1	2 ± 0.5	2 ± 0.5
*Candida parapsilosis* RM	8 ± 0.5	8 ± 0.5	2 ± 0.5	2 ± 0.5

MIC and MLC values represent the mean ± SD of three independent experiments. MIC: Minimum Inhibitory Concentrations. MLC: Minimum Lethal Concentrations.

**Table 3:** In vitro anti-*T. vaginalis* activity of essential oils from the leaves of *P. trimera* and *L. littoralis.*

Time	*P. trimera* Oil			*L. littoralis* Oil		
	IC_50_	IC_90_	MLC	IC_50_	IC_90_	MLC
**1 h**	2	4	8	2	8	16
**4 h**	0.5	1	2	1	4	8
**24 h**	0.03	0.06	0.12	0.12	0.25	0.5
**48 h**	0.016	0.03	0.06	0.03	0.06	0.12

Data represent mean values for two independent experiments. IC_50_: The concentration that causes 50% Trichomonas growth inhibition (% *v/v*). IC_90_: The concentration that causes ≥ 90% Trichomonas growth inhibition (% *v/v*). MLC: The concentration that causes the death of 100% Trichomonas (% *v/v*).
